# A Case of Penicillin-Resistant* Veillonella* Prosthetic Joint Infection of the Knee

**DOI:** 10.1155/2016/7171947

**Published:** 2016-12-05

**Authors:** Claudia R. Libertin, Joy H. Peterson, Mark P. Brodersen, Tamara Huff

**Affiliations:** ^1^Division of Infectious Diseases, Mayo Clinic Health System (MCHS-W), Waycross, GA, USA; ^2^Departments of Pathology and Laboratory Medicine, Mayo Clinic Health System-Waycross, Waycross, GA, USA; ^3^Division of Infectious Diseases, Mayo Clinic, Jacksonville, FL, USA; ^4^Department of Orthopedics, Mayo Clinic Health System-Waycross, Waycross, GA, USA; ^5^Department of Orthopedic Surgery, Mayo Clinic, Jacksonville, FL, USA

## Abstract

*Veillonella *sp. and* V. dispar* are emerging pathogens. This is the third case of a monomicrobial* Veillonella* sp. prosthetic joint infection (PJI) among knees and hips; this is the second prosthetic knee infection described. The infection was treated with a 2-stage procedural approach combined with 6 weeks of ceftriaxone with excellent clinical response. There was no relapse in 2 years of follow-up care. This case exemplifies the importance of incubating anaerobic cultures for at least 7 days to grow some anaerobic pathogens.

## 1. Introduction

Diagnosing prosthetic joint infections takes a disciplined approach by orthopedic surgeons to make a bacterial diagnosis [1]. Prosthetic joint infections due to* Veillonella *sp. as the sole pathogen remain rarely reported.* Veillonella *sp. are small, strictly anaerobic gram-negative cocci that are found in the oral, respiratory, genitourinary, and intestinal tracts [2]. Infections such as bacteremia [3], meningitis [4], endocarditis [5], osteomyelitis [6, 7], and prosthetic joint infections (PJI) [8, 9] have been described. This is only the second prosthetic knee infection [9] reported due solely to* Veillonella* sp. and only the third prosthetic joint infection [8] including hips. In addition to supporting that* Veillonella* acts as a pathogen, this case exemplifies the need for microbiology laboratories to follow standard microbiologic practices such as incubating anaerobic cultures for a minimum of 7 days.

Informed consent was obtained from the patient to report this case.

## 2. Case Report

**Figure 1:**
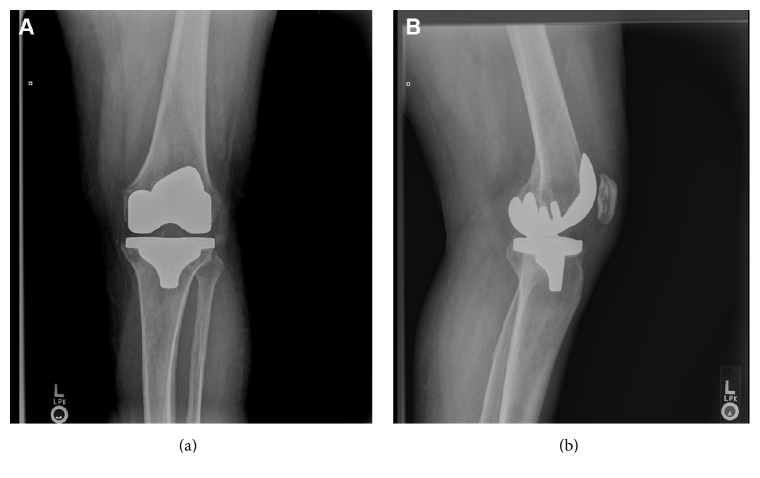
Anterior-posterior (a) and lateral (b) radiographs of left primary total knee arthroplasty from initial clinic presentation.

A 61-year-old man with well-controlled, noninsulin-dependent diabetes and a history of an uncomplicated left total knee arthroplasty implanted 2 years before presented with a 2-month history of acute knee pain with progressive swelling, erythema, and an inability to ambulate. He had no fevers, chills, or night sweats. He received no empiric antibiotics. Besides a swollen and warm left knee, he had extensive caries and gingivitis on examination. His X-rays revealed a well fixed total knee arthroplasty (Figure 1).

Two aspirates of the knee done in a clinic at Mayo Clinic Health System-Waycross (MCHS-W; formerly Satilla Regional Medical Center) revealed brown, purulent-appearing fluid (Table 1). White blood cell count from the initial aspiration was 72,696 cell/uL with 88% polys. Gram stain showed white blood cells, but no organisms were seen. Only cultures were requested on the second knee aspirate done in the clinic. The clinic aspirate samples were submitted to a national commercial microbiology laboratory and not MCHS-W hospital's microbiology laboratory. Cultures were reported to have no growth on two separate occasions (Table 1). Because of persistent pain, the orthopedic surgeon admitted the patient to MCHS-W hospital to remove the prosthesis, place a static antibiotic spacer, and submit 4 operative tissue specimens to the Department of Laboratory Medicine and Pathology. His white blood cell count was 15.8 × 10^5^/*μ*L, erythrocyte sedimentation rate was 100 mm/hour, and C-reactive protein was 138 mg/L. The patient was started on parenteral vancomycin and ciprofloxacin empirically for a presumed diagnosis of culture-negative PJI (CN PJI) by the orthopedic team. While hospitalized, an infectious disease consultation was obtained. Prior negative aerobic cultures and inadequate incubation times for anaerobic cultures raised suspicion that an anaerobic organism is the pathogen. The consultant confirmed with the microbiology laboratory that an anaerobic culture had indeed been submitted and requested that the anaerobic cultures be held for 10 days. It was discovered that the MCHS-W hospital laboratory, which is a rural community microbiology laboratory, only held anaerobic cultures for 2 days unless otherwise requested.

All operative samples were plated on Remel media (Remel, Lenexa, KS). Blood agar (BAP) and chocolate agar were incubated at 35°C in 5% CO_2_; MacConkey agar and thioglycolate broth were incubated at 35°C in ambient air. BAP and phenyl ethyl alcohol agar were incubated anaerobically with the Anoxomat System (Advanced Instruments, INC; MART Microbiology, BV, Drachten, Netherlands). Bactec Pediatric Plus/F bottles (Becton Dickinson, Sparks, MD) were not inoculated. All original aerobic and anaerobic plates showed no growth at 2 days. On day 4, a gram stain performed directly from the thioglycolate broth of all 4 specimens revealed small gram-negative cocci. All 4 thioglycolate broths were plated to BAP CO_2_ and BAP anaerobic. All BAP CO_2_ subcultures were no growth. All BAP anaerobic subcultures grew small round translucent colonies that stained as gram-negative cocci. The Remel RapID ANA II System identified the organism as* Veillonella* sp. Because* Veillonella* sp. yield a negative biochemical profile except for the production of catalase and nitrate reduction, the bacterial isolate was sent to Mayo Medical Laboratories (MML, Rochester, MN) for confirmation. The Bruker Biotyper was used to identify the pathogen by MALDI-TOF (Matrix Assisted Laser Desorption Ionization-Time of Flight) methodology. Bruker MALDI-TOF identified the isolate as* Veillonella parvula* to an acceptable level of 2.173 (an acceptable identification to the genus level with a confidence level is as low as 1.70). The isolate was further speciated by real time polymerase chain reaction (PCR) on the microbial identification kit from Applied Bio systems. The master-mix of primers/probes is made in-house; all components are purchased individually. Primers and probes are purchased from Integrated DNA Technologies, INC (IDT, Coralville, Iowa). MML performs dual PCR with Applied Biosystems and then performs 16S rRNA sequencing with IDT primers and probes. 16S rRNA sequencing was done at Mayo Medical Laboratory. After performing 16S rRNA sequencing on the* Veillonella* isolate differentiation among 3 possible species:* V. parvula*,* V. dispar*, and* V. rodentium* could not be determined. All three comparison strains were ATCC reference strains. The percentages of similarity were 99.54, 99.49, and 99.25, respectively.

Susceptibilities were performed by Etest methodology (bioMerieux, Durham, NC) on* Brucella*-based agar at MML and are reported in Table 2.

**Figure 2:**
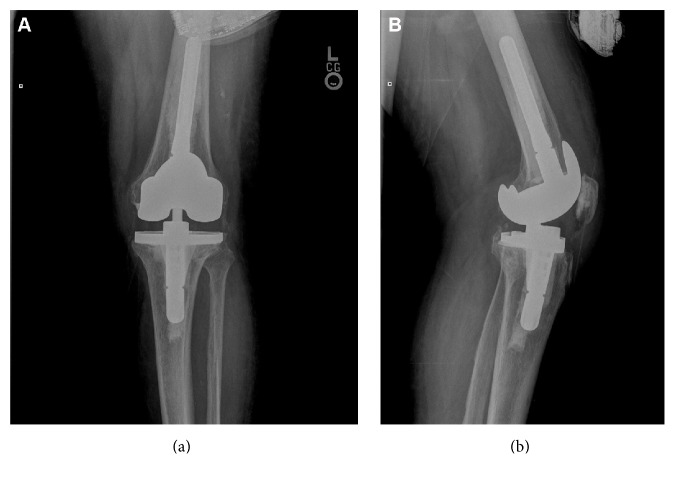
Anterior-posterior (a) and lateral (b) radiographs after second stage of reimplantation of cemented revision left total knee arthroplasty.

The patient received 6 weeks of parenteral ceftriaxone in the community following the removal of the prosthesis. During antibiotic therapy, extensive dental work and extractions were performed since his dental caries, dental abscesses, and gingivitis could have been a potential source of the infection. After completion of the therapy and an antibiotic holiday, interventional radiology attempted unsuccessfully to aspirate the knee. At reimplantation, frozen bone sections were negative for bacteria and acute inflammation.  Figure 2 shows the reimplanted knee arthroplasty. The patient's recovery from reimplantation was uneventful. Two years later, he has not had a relapse of PJI in that knee.

## 3. Discussion

Marchandin et al. [9] described the first report of a monomicrobial prosthetic joint infection caused by* Veillonella dispar* in a prosthetic knee in 2001. All* Veillonella* sp. reported to cause monomicrobial PJI are listed and compared in Table 3. The two PKIs of the knees had similar clinical presentations of joint swelling associated with pain and functional incapacity. Our patient did not have evidence of prosthesis loosening radiographically (Figure 1), as the other cases did. Our patient's isolate was identified by MALTI-TOF methodology followed by real-time PCR and by 16S rRNA sequencing with IDT primer/probes to confirm the pathogen, but determination of the species was not made. Our patient received 6 weeks of ceftriaxone therapy after removal of the prosthesis. Two years later, there were no manifestations of a relapse of infection. Zaninetti-Schaerer et al. [8] described a monomicrobial* Veillonella* sp. prosthetic hip infection which was associated with bacteremia. That infection was treated with 3 weeks of ceftriaxone followed by amoxicillin and imipenem for a total of 3 months. Lifelong oral clindamycin without prosthesis was prescribed. Ongoing monitoring of the patient post treatment was not documented. Of these three monomicrobial prosthetic joint infections, our patient did well with a standard 6 weeks of ceftriaxone therapy after prosthesis removal. In all three PJIs from* Veillonella* sp., dental sources were suspected but never definitively proven.

The susceptibility profile of our isolate is typical of those reported in recent literature [10–12]; most strains of* Veillonella* sp. are now penicillin resistant (Table 2). Resistance to vancomycin is an innate resistance. Manchandin et al. isolate was penicillin susceptible [9]. Susceptibilities on the isolate from the total hip arthroplasty (THA)* Veillonella* sp. infection were not reported [8].

Unfortunately, our patient was initially falsely diagnosed as a CN PJI based on the fact that the orthopedic surgeon had ordered anaerobic cultures when the patient was seen in the clinic. Two knee aspirates were performed but sent to a commercial laboratory from our clinic instead of the hospital microbiology laboratory. Cultures revealed no growth (Table 1). CN PJI can result from sampling practices by surgeons, failure to order anaerobic cultures, methodologies used in the microbiology laboratory, and most commonly prior use of antimicrobial therapy within the 3 months before cultures are obtained [13]. The inappropriate empiric initiation of vancomycin and ciprofloxacin occurred initially because the surgeon had ordered anaerobic cultures and even had requested from the national commercial laboratory that they be held for 7 days with the cultures reported as negative. The inadequate incubation times for anaerobic organism detection resulted in the reporting of false negative results. The requesting of anaerobic cultures, submitting 4 tissue specimens, and incubating anaerobic cultures for a minimum of 7 days are vital in making a bacteriologic diagnosis [1, 14]. It is critical to confirm with the microbiology laboratories used to process and identify patients' organisms that the incubation standards for isolating anaerobic cultures are followed. Even if the anaerobic incubation time had not been extended by the infectious disease physician at MCHS-W, the organism would have been recovered from aerobic cultures in that the organism was growing in thioglycolate broth on day 4. This case reveals the importance of clinicians being diligent in confirming that each step in the process of identifying a bacterial isolate is done according to the standard of practice in that discipline.

In summary, we report the second case of monomicrobial* Veillonella* sp. prosthetic knee infection successfully treated with a 2-stage orthopedic approach and 6 weeks of ceftriaxone therapy. This case supports* Veillonella* sp. as an emerging pathogen causing monomicrobial PJIs. It also exemplifies the importance of all microbiology laboratories to analyze an anaerobic sample properly utilizing adequate microbiological practices as advised by national standards and for clinicians to confirm that the standards are done.

## Figures and Tables

**Table 1:** Impact of anaerobic incubation time on growth detection.

Knee sample	Fluid appearance	Culture Orders	Laboratory	Duration of incubation	Microbiologic results
Arthrocentesis #1	Brown, purulent	Aerobic & anaerobic cultures	National commercial lab	48 hours	No growth
Arthrocentesis #2	Brown, purulent	Aerobic culture hold for 7 days	National commercial lab	72 hours	No growth
Synovial operative tissue ×4	No description	Aerobic & anaerobic cultures	MCHS-Waycross	10 days	*Veillonella* sp.

MCHS: Mayo Clinic Health System.

**Table 2:** *Veillonella* isolate.

Antibiotic	MIC (mcg/mL)	CLSI guidelines		Interpretation
		Susceptible	Resistant	
Penicillin	8	≤0.5	>1	Resistant
Clindamycin	≤0.5	≤2	>4	Susceptible
Metronidazole	1	≤8	>16	Susceptible
Ceftriaxone	8	≤16	>32	Susceptible
Ciprofloxacin	0.12	No interpretive criteria given		No interpretation

CLSI: Clinical Laboratory Standards Institute; MIC: minimal inhibitory concentration tested by Etest methodology performed by Mayo Medical Laboratory.

**Table 3:** Monomicrobial *Veillonella* prosthetic joint infections.

Case report	Year	PJI site	Symptoms	X-ray	Organism	E test sensitivities	Surgical	Medical treatment	Follow-up evaluation
Marchandin et al. [9]	2001	Knee	Pain & swelling functional incapacity	Prosthetic loosening	*Veillonella dispar*	Penicillin (S) Amoxicillin/clavulanate (S) clindamycin (S)	2-stage procedure	Amoxicillin + rifampicin × 6 months	6 months
Zaninetti-Schaerer et al. [8]	2004	Hip	Pain & functional incapacity	Prosthetic loosening	*Veillonella* sp.	Not reported	Retained prosthesis	Ceftriaxone 3 wks, then ampicillin, followed by imipenem, for a total of 3 months; lifelong po clindamycin	Not reported
Current	2016	Knee	Pain & swelling functional incapacity	No prosthetic loosening	*Veillonella *sp.	Penicillin (R)	2-stage procedure	Ceftriaxone 2 gm qd × 6 wks	2 years

PJI: prosthetic joint infection, R: resistant, and S: sensitive.
